# Six-Gene Signature for Differential Diagnosis and Therapeutic Decisions in Non-Small-Cell Lung Cancer—A Validation Study

**DOI:** 10.3390/ijms25073607

**Published:** 2024-03-23

**Authors:** Radoslaw Charkiewicz, Anetta Sulewska, Piotr Karabowicz, Grzegorz Lapuc, Alicja Charkiewicz, Marcin Kraska, Joanna Pancewicz, Malgorzata Lukasik, Miroslaw Kozlowski, Rafal Stec, Dominika Ziembicka, Weronika Piszcz, Wojciech Miltyk, Wieslawa Niklinska

**Affiliations:** 1 Center of Experimental Medicine, Medical University of Bialystok, 15-369 Bialystok, Poland; 2 Department of Clinical Molecular Biology, Medical University of Bialystok, 15-269 Bialystok, Poland; anetta.sulewska@umb.edu.pl (A.S.); marcin.kraska92@gmail.com (M.K.); weronikapiszcz@gmail.com (W.P.); 3 Biobank, Medical University of Bialystok, 15-269 Bialystok, Poland; piotr.karabowicz@umb.edu.pl; 4 Department of Thoracic Surgery, Medical University of Bialystok, 15-269 Bialystok, Poland; lapucg@gmail.com (G.L.); miroslaw.kozlowski@umb.edu.pl (M.K.); 5 Department of Analysis and Bioanalysis of Medicines, Medical University of Bialystok, 15-089 Bialystok, Poland; alicja.charkiewicz@umb.edu.pl (A.C.); wojciech.miltyk@umb.edu.pl (W.M.); 6 Department of Medical Pathomorphology, Medical University of Bialystok, 15-269 Bialystok, Poland; 7 Department of Histology and Embryology, Medical University of Bialystok, 15-269 Bialystok, Poland; joanna.pancewicz@umb.edu.pl (J.P.); malgorzata.lukasik@umb.edu.pl (M.L.); 8 Department of Oncology, Medical University of Warsaw, 02-091 Warsaw, Poland; rafal.stec@uckwum.edu.pl; 9 Department of Public Health, Medical University of Bialystok, 15-295 Bialystok, Poland; dmziembicka@gmail.com

**Keywords:** NSCLC subtyping, SCC, ADC, gene expression, qPCR, personalized medicine, prognosis, validation study

## Abstract

Non-small-cell lung cancer (NSCLC) poses a challenge due to its heterogeneity, necessitating precise histopathological subtyping and prognostication for optimal treatment decision-making. Molecular markers emerge as a potential solution, overcoming the limitations of conventional methods and supporting the diagnostic–therapeutic interventions. In this study, we validated the expression of six genes (*MIR205HG*, *KRT5*, *KRT6A*, *KRT6C*, *SERPINB5*, and *DSG3*), previously identified within a 53-gene signature developed by our team, utilizing gene expression microarray technology. Real-time PCR on 140 thoroughly characterized early-stage NSCLC samples revealed substantial upregulation of all six genes in squamous cell carcinoma (SCC) compared to adenocarcinoma (ADC), regardless of clinical factors. The decision boundaries of the logistic regression model demonstrated effective separation of the relative expression levels between SCC and ADC for most genes, excluding *KRT6C*. Logistic regression and gradient boosting decision tree classifiers, incorporating all six validated genes, exhibited notable performance (AUC: 0.8930 and 0.8909, respectively) in distinguishing NSCLC subtypes. Nevertheless, our investigation revealed that the gene expression profiles failed to yield predictive value regarding the progression of early-stage NSCLC. Our molecular diagnostic models manifest the potential for an exhaustive molecular characterization of NSCLC, subsequently informing personalized treatment decisions and elevating the standards of clinical management and prognosis for patients.

## 1. Introduction

Using advanced methods to explore new genetic and epigenetic markers represents a significant step forward in distinguishing between squamous cell carcinoma (SCC) and adenocarcinoma (ADC) compared to traditional examination methods [1,2]. While histopathology is considered the gold standard, it faces challenges such as inconsistent results from immunohistochemical staining (IHC), variations in tumor characteristics, and difficulties in interpreting staining results [2]. This variability in histological subclassifications of non-small-cell lung cancer (NSCLC) is often due to differences in sampling and evaluation protocols [2]. In about 15–20% of tissue biopsy cases, it is difficult to determine the exact NSCLC subtype using traditional methods, leading to a diagnosis of non-small-cell carcinoma without specifying the subtype (NOS) [3].

In addition to challenges in the precise subtyping of NSCLC, many patients are diagnosed at advanced stages, limiting treatment options [4]. Despite surgical intervention, up to 30% of stage I patients experience recurrences within 5 years [5]. The use of adjuvant chemotherapy in stage I cases remains controversial, but it may benefit high-risk patients identified through molecular categorization. Therefore, there is a need to identify reliable predictors of relapse to improve the management of early-stage NSCLC.

Personalized oncology emphasizes the importance of the precise histopathological classification of NSCLC alongside the examination of somatic mutations [6,7,8]. Tailored therapies, especially for non-squamous NSCLC with EGFR mutations, and the use of monoclonal antibodies like bevacizumab and ALK inhibitors have shown promising results in specific subtypes [9,10,11,12]. Recent findings from trials like ADAURA suggest improved survival outcomes for patients with EGFR-mutated non-squamous NSCLC, highlighting the evolving landscape of precision oncology [13]. Given the importance of targeted and immunotherapeutic treatments, there is a critical need for precise diagnostic methods to accurately classify NSCLC histologically. This classification is crucial for identifying the most suitable treatment options for patients.

In our previous research, we identified potential biomarkers for NSCLC, such as hsa-miR-205 and hsa-miR-21, which can distinguish between ADC and SCC with high accuracy [2]. Using miRNA sequencing, we developed a 17-miRNA signature with excellent discriminatory power for subtype differentiation within NSCLC [14]. Additionally, a carefully selected 14-lncRNA signature showed proficiency in detecting NSCLC and classifying subtypes [15]. We also identified a serum-based signature of the top 15 miRNAs, which demonstrated exceptional discriminatory ability [16].

Building on previous screening studies using gene expression microarrays, we created a 53-gene signature with impressive accuracy in distinguishing between SCC and ADC [17]. Six genes from this signature, including KRT5, KRT6A, KRT6C, DSG3, SERPINB5, and MIR205HG, showed the most discriminatory capabilities [18]. Our main goal was to analyze the expression levels of these six genes using real-time PCR in tumor tissues from a well-characterized cohort of NSCLC patients with completely resected tumors and to develop an objective molecular test for NSCLC subtype differentiation.

This study represents a significant advancement in improving NSCLC diagnosis and subtyping precision. By identifying new biomarkers and developing a gene expression diagnostic test, we aim to improve patient care by guiding personalized treatment strategies and complementing traditional histopathological examinations. We believe our research will lead to more effective management of NSCLC in the future.

## 2. Results

### 2.1. Patients’ Characteristics

In a study involving 140 surgically treated non-small-cell lung cancer patients who underwent complete tumor resection, 56.42% were diagnosed with squamous cell carcinoma (SCC), while 43.57% had adenocarcinoma (ADC). The patient cohort consisted of 33 women (23.57%) aged 50–78 and 107 men (76.43%) aged 40–81, with an average age of 63.33. Squamous cell carcinoma was more prevalent in men, accounting for 61.68% of the male patient group, whereas adenocarcinoma was predominant in women, constituting 60.61% of the female patient group. Cancer staging using the TNM classification revealed that 46.43% had stage I cancer, 47.14% had stage II, and 5.71% had stage IIIA. The stage of one patient was unknown. In terms of clinical characteristics, 38.57% of patients experienced disease recurrence after complete tumor resection during a minimum 3-year follow-up, while 58.57% had no recurrence. Data on cancer recurrence were missing for 2.86%. Detailed patient characteristics can be found in Table 1.

### 2.2. Differences in the Expression Profiles of Validated Genes between the Histological Subtypes of NSCLC (SCC vs. ADC)

**Figure 1:**
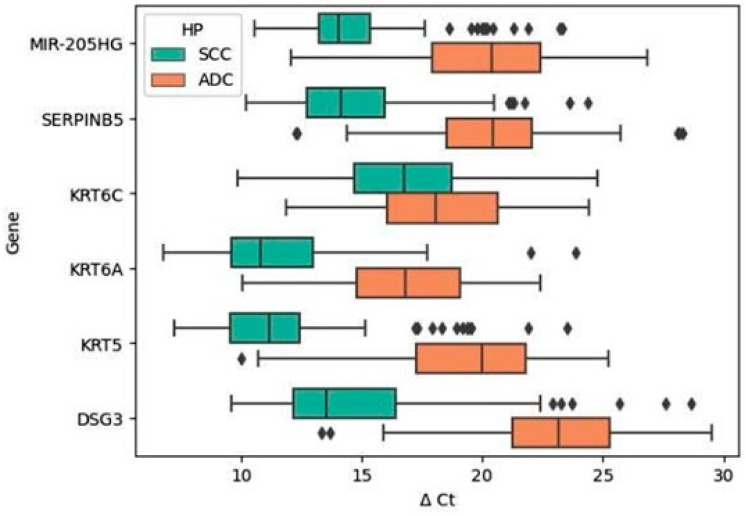
The divergence in ∆Ct distributions across all genes between the SCC and ADC subtypes within the tumor tissue. Diamonds indicate the outlier patients in the data. ∆Ct values are inversely proportional to the direction of relative gene expression.

The expression levels of all six validated genes showed a significant association with the histopathological subtype of NSCLC. In SCC tumors, in contrast to ADC tumors, we observed increased expression levels for all validated genes, namely, *MIR205HG*, *SERPINB5*, *KRT6C*, *KRT6A*, *KRT5*, and *DSG3*. The analysis revealed noteworthy findings, emphasizing statistically significant differences in the expression levels of all these genes between SCC and ADC. Notably, *KRT6C* exhibited the weakest statistical significance, although it remained significant (Figure 1 and Table 2). Furthermore, we noted a consistent pattern in the relative expression levels of the six validated genes, aligning with those observed in the initial screening study utilizing gene expression microarray technology [17].

### 2.3. Relationships between Expression Levels of Validated Genes and Clinicopathological Characteristics of NSCLC Patients

The expression levels of transcripts for all six genes, namely, *MIR205HG*, *SERPINB5*, *KRT6C*, *KRT6A*, *KRT5*, and *DSG3*, were found to be strongly linked to the histological subtype of NSCLC. Notably, the relative expression levels of the analyzed genes between SCC and ADC subtypes consistently showed statistically significant differences, regardless of the status of other clinicopathological features, including sex, age, TNM staging, and postoperative disease recurrence among the patients. Only the gene expression level for *KRT6C* exhibited a weaker correlation. This suggests a distinct association between these selected genes and the histological subtype of NSCLC, independent of other clinical parameters (Table S1).

### 2.4. Visualizing Decision Boundary for Logistic Regression Models of Individual Validated Genes

**Figure 2:**
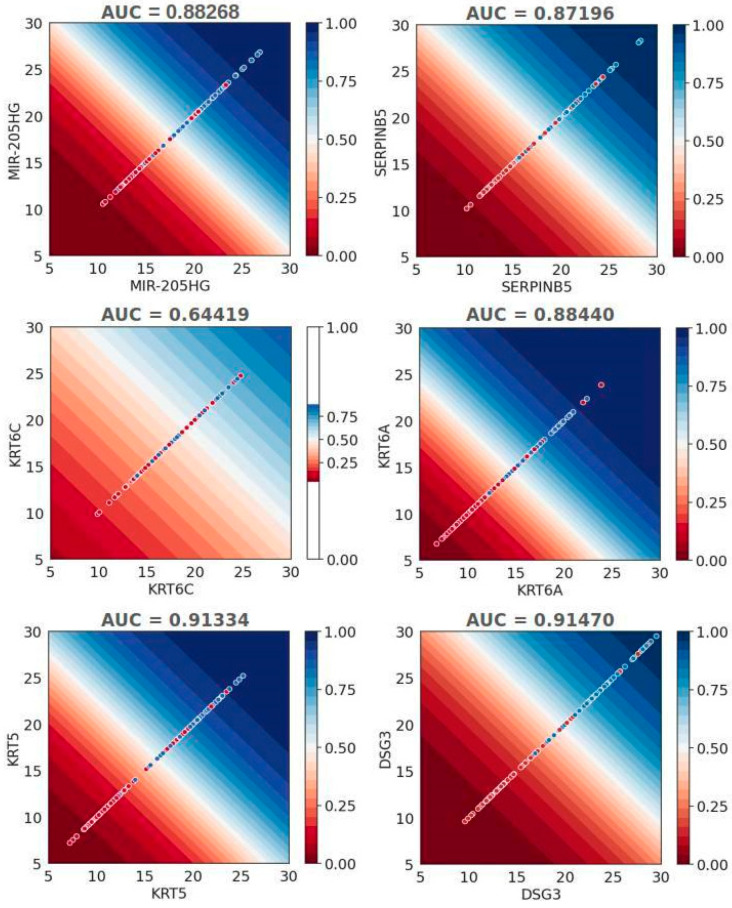
The decision boundary and AUC values for logistic regression models. Each dot represents a sample’s true ΔCt values, with red indicating SCC and blue indicating ADC histologies. The decision boundary comprises points where the probability of assigning a sample to one of the class/histology subtypes is precisely 0.5. The gradual surface corresponds to the probability of assigning a ΔCt value to the appropriate class. Points on the red surface are predicted to belong to the SCC class, while points on the blue surface are predicted to belong to the ADC class.

To illustrate the discrimination between NSCLC subtypes, we developed six logistic regression models based on the relative expression levels of specific genes. The logistic regression decision boundary indicates a probability of 0.5 for assigning a sample to one of the class/histology subtypes. In the plots, a clear white line represents this boundary, intersecting each probability allocation for the histologies. The plot surface vividly displays probabilities, with blue (0.5 to 1.0) indicating a higher probability assignment to ADC and red (0.5 to 0.0) indicating a higher probability assignment to SCC. These six plots, each corresponding to an analyzed gene, illustrate how the decision boundary separates NSCLC subtype assignations. Data points on the plots represent the samples’ ∆Ct values, with red indicating squamous cell carcinoma (SCC) and blue indicating adenocarcinoma (ADC). Notably, a clear separation of ∆Ct values by histopathological types was observed in most cases, except for KRT6C (Figure 2).

### 2.5. Gene Expression Value in NSCLC Subtyping Based on Logistic Regression and Gradient Boosting Decision Tree Models

**Figure 3:**
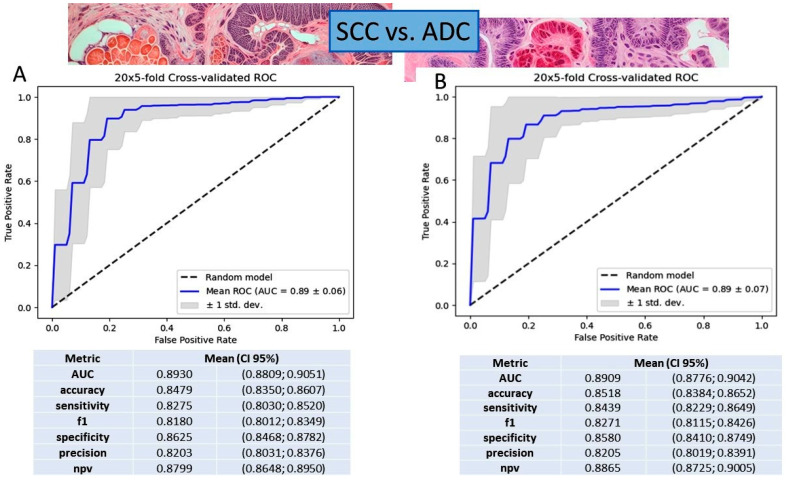
Model metrics for (**A**) logistic regression and (**B**) gradient boosting decision tree classifiers based on all 6 genes together. The figure includes the mean receiver operating characteristic (ROC) curve with standard deviation (SD) and the mean area under the curve (AUC) for both classifiers. Additionally, it provides the mean and 95% confidence interval (CI) of accuracy, F1 score metrics, AUC, specificity, sensitivity, and precision, specifically focused on the classifiers’ performance in differentiating squamous cell carcinoma (SCC) from adenocarcinoma (ADC).

We assessed two different methods, the logistic regression classifier and gradient boosting decision tree, using all six validated genes together to distinguish between non-small-cell lung cancer (NSCLC) subtypes. Both methods proved effective, showing similar performance metrics. The logistic regression model performed well, achieving an AUC of 0.8930, an accuracy of 0.8479, a sensitivity of 0.8275, a precision of 0.8203, a specificity of 0.8625, and an NPV of 0.8799. Similarly, the gradient boosting model also demonstrated high performance, with an AUC of 0.8909, an accuracy of 0.8518, a sensitivity of 0.8439, a precision of 0.8205, a specificity of 0.8580, and an NPV of 0.8865. Upon comparison, we noted the following subtle differences: logistic regression had a slightly higher AUC and specificity, while gradient boosting performed better in sensitivity and NPV. Choosing between the two models may depend on specific priorities or trade-offs based on the intended application and the unique contributions of the selected genes (see Figure 3).

**Figure 4:**
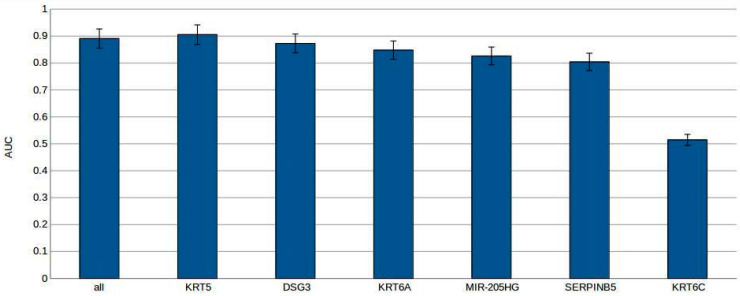
The mean AUC and 95% CI for gradient boosting decision tree models, both when considering all six genes together and individually for each gene.

We also assessed the performance of the gradient boosting decision tree classifier using individual genes, in comparison to the combination of all genes together as features, to predict sample classes/histologies. The area under the curve (AUC) served as our metric to quantify the predictive strength of each classifier. In Figure 4, AUC values are depicted for classifiers employing either all six genes together or individual genes (*KRT5*, *DSG3*, *KRT6A*, *MIR-205HG*, and *SERPINB5*) as features. Classifiers utilizing *KRT5* alone, *DSG3* alone, *KRT6A* alone, *MIR-205HG* alone, *SERPINB5* alone, or the combined set of all six genes exhibit high AUC values near 0.9, signifying robust predictive capability. However, the classifier relying on *KRT6C* alone demonstrates a significantly lower AUC value of 0.51, indicating diminished predictive performance. These findings suggest that *KRT5*, *DSG3*, *KRT6A*, *MIR-205HG*, and *SERPINB5* serve as reliable features for predicting sample histology subtypes, while *KRT6C* does not share similar predictive efficacy (Figure 4).

### 2.6. Prognostic Significance of Validated Gene Expression in NSCLC Patients

In exploring the potential of genes as prognostic factors in NSCLC, we investigated the influence of gene expression levels in primary tumors on the recurrence status of the patients post-surgical treatment. A comprehensive comparison between patients experiencing recurrence within three years and those without revealed an absence of statistically significant genes within the study set (Table S2). The results are consistent with our previous findings from the analysis of the 53-gene signature [17].

### 2.7. Gene Ontology Analyses

Gene Ontology (GO) analyses of the validated genes show significant roles in various cellular components, biological processes, and molecular functions. DSG3 and SERPINB5 are located in the cornified envelope and extracellular space, aiding in intermediate filament organization. KRT6A and KRT5, components of keratin filaments and the intermediate filament cytoskeleton, participate in filament organization, keratinization, and epidermal cell differentiation within the skin’s epidermis. Similarly, KRT6C, localized in keratin filaments, aids in filament organization and the differentiation of keratinocytes and epidermal cells within the skin’s epidermis (Table 3).

## 3. Discussion

In this study, we formulated robust models adept at discerning non-small-cell lung cancer (NSCLC) subtypes, thereby fortifying routine diagnostic procedures. This augmentation in diagnostic precision holds the potential to influence treatment qualification and subsequently yield therapeutic advantages. To achieve this, we validated the expression profiles of six specific genes (*MIR205HG*, *KRT5*, *KRT6A*, *KRT6C*, *SERPINB5*, and *DSG3*) as robust candidates for histological discrimination between NSCLC subtypes. Additionally, we assessed the prognostic significance of these genes. The selection of these top genes was guided by a 53-gene signature developed by our team, demonstrating significance in lung cancer classification [17]. Our results underscore a consistent upregulation of all six genes in squamous cell carcinoma (SCC) compared to adenocarcinoma (ADC). Notably, these genes, whether used individually (excluding *KRT6C*) or collectively (including *KRT6C*), prove to be independent factors for precise NSCLC subtyping. Additionally, GO analyses of validated genes highlight their roles in cellular components, biological processes, and molecular functions. DSG3 and SERPINB5 aid in intermediate filament organization within the cornified envelope and extracellular space. KRT6A, KRT5, and KRT6C contribute to filament organization, keratinization, and epidermal cell differentiation in the skin’s epidermis. Moreover, the clear statistical significance obtained for the analyzed genes reaffirms the earlier results, despite methodological differences. Previous research relied on a screening method, the gene expression microarray, while in this study, we employed a quantitative method, qPCR.

Significant differences in gene expression levels were observed between SCC and ADC when analyzing all six validated genes together, employing the gradient boosting decision tree and logistic regression classifiers. Both classifiers demonstrated strong predictive capabilities, with nuanced differences in performance metrics. The gradient boosting decision tree model exhibited high discriminative ability (AUC: 0.8909) and accuracy (0.8518), with notable sensitivity (0.8439) and precision (0.8205). Similarly, the logistic regression model achieved high performance metrics (AUC: 0.8930, accuracy: 0.8479, sensitivity: 0.8275, precision: 0.8203). While the models showed subtle differences, the choice between them may depend on specific priorities or trade-offs. Furthermore, the evaluation of classifier performance, whether considering individual genes or a combination of all six genes, demonstrated robust predictive capabilities, with AUC values nearing 0.9 for the majority of gene sets. Notably, *KRT6C* alone exhibited diminished predictive performance (AUC: 0.51), emphasizing the importance of the other genes in sample class prediction. Moreover, the decision boundary of the logistic regression underscored a distinction in ΔCt values among histopathological subtypes for all individual genes, except for *KRT6C*.

While numerous publications have explored gene expression signatures for NSCLC subtyping, none have undertaken an exploration of our distinctive six-gene signature to date. Li et al. [19] successfully identified 20 genes specific to adenocarcinoma in situ, achieving an impressive AUC of 0.98. Niemira et al. [20] delved into the identification of 7720 and 5856 genes with diverse expression (DEGs) in squamous cell carcinoma (SCC) and adenocarcinoma (ADC) of the lungs, respectively, when compared to the control group. Notably, certain genes, such as CTLA4, MZB1, NIP7, and BUB1B in ADC, as well as GNG11 and CCNB2 in SCC, were found to be associated with tumor size, adding a crucial dimension to our understanding of lung cancer biology. Meanwhile, Song et al. [21] developed a 14-gene lncRNA predictive prognostic model capable of classifying patients into high- or low-risk groups, with an AUC of 0.705. In our previous study [15], we were also centered on lncRNAs and developed two predictive models for the early diagnosis of NSCLC. These models demonstrated that the set of 14 lncRNAs accurately distinguished between cancerous and noncancerous lung tissues (AUC value of 0.98 ± 0.01) and NSCLC subtypes (AUC value of 0.84 ± 0.09). The streamlined logistic regression model, utilizing four parameters (∆Ct LINC00673, ∆Ct AFAP1-AS1, Ct LINC0026, and Ct SOX2-OT), demonstrated enhanced diagnostic capability for subtype discrimination, yielding an AUC value of 0.88 ± 0.07. 

Interestingly, a subset of studies focused on identifying molecular diagnostic markers for the precise stratification of NSCLC subtypes [22,23,24,25] correlates with our earlier research, where we developed a 53-gene signature [17]. From this signature, we specifically chose six genes for validation in the present study. Girard et al. [22] developed a 62-gene signature (42 for ADC/SCC and 20 for nonmalignant/lung cancer discrimination), achieving high accuracies (93–95%). Our slightly superior signature reached 100% correct prediction for ADC samples [17]. Hou et al. [23] and Wilkerson et al. [24] presented 75- and 51-gene signatures, with accuracies of 83% and 84%, respectively. Our group identified and validated a histology-associated gene signature, ensuring 100% correct classification of all ADC tumors, crucial for targeted therapies limited to specific mutations [17]. Liu et al. [25] defined differentially expressed genes and 16 pathways for ADC and SCC. ADC exhibited more prevalence in immune response-related pathways, aligning with our emphasis on immune processes in ADC histology.

It is essential to highlight a distinctive aspect of our study design, which differs significantly from existing approaches. We focused on subtype differentiation by exclusively comparing gene expressions in tumors, considering both validated and reference genes. In this approach, we deliberately excluded healthy tissue from the analysis to mitigate individual variations. Discrepancies in our results compared to the literature data can be attributed to variations in experimental layouts (tumor tissue vs. normal tissue), the use of diverse biological materials (tissue, serum, plasma, and cell culture), the application of different technologies (qPCR, gene expression microarray, NGS, etc.), the limited study of certain genes in NSCLC, and the necessity to draw insights from other cancers where expression patterns may vary.

Within the study cohort, we noted a significant increase in *MIR205HG* expression in SCC in comparison to ADC. *MIR205HG* is a long non-coding RNA (lncRNA) with a length of 895 nucleotides. Its expression has been reported to vary across different cancer types, being downregulated in esophageal cancer [26], upregulated in hepatoblastoma [27] and cervical cancer [28], and positively correlated with head and neck squamous cell carcinoma, particularly in cases with *TP53* mutations [29]. However, in the context of SCC, the existing literature presents conflicting results, some suggesting downregulation while others indicating upregulation in comparison to healthy tissue [30,31].

*MIR205HG* plays a multifaceted role in various cellular pathways. For instance, in hepatoblastoma, it activates the MAPK signaling pathway through competitive binding to miR-514a-5p, effectively acting as a “sponge” for miR-205-5p and thereby activating the PI3K/AKT pathway [27]. In head and neck squamous cell carcinoma, *MIR205HG* inactivates miR-590-3p, leading to increased expression of cyclin B, cdk1, and YAP [29]. In lung squamous cell carcinoma, it promotes proliferation, migration, and tumor progression by modulating the expression of Bcl-2 and Bax [32], enhancing ITGB8 expression [30], and binding to miR-299-3p, resulting in elevated MAP3K2 expression [31].

Importantly, *MIR205HG* serves as the host gene for hsa-miR-205, a microRNA that we have previously investigated and demonstrated to be associated with distinctive features of squamous histology in NSCLC. [2]. Larzabal et al. [33] unveiled a novel signaling pathway involving membrane-anchored proteins (ITGα5 and TMPRSS4) that promote tumor growth, metastasis, and migration through miR-205. Interestingly, our 53-gene signature, from which we selected genes for validation in this study, included *TMPRSS11D*, a gene from the same family as *TMPRSS4*. Consistent with Larzabal’s findings, our 53-gene signature showed elevated expression of both *MIR205HG* and *TMPRSS11D* in SCC compared to ADC samples [17]. Key genes in our signature, such as *CLDN3*, *TMPRSS11D*, *SERPINB5*, and *FGFBP1*, could be promising immunostains for the pathology-based differential diagnosis of NSCLC subtypes.

*KRT6A*, another gene of interest, exhibited significantly higher expression in SCC when compared to ADC. Keratin 6A is a type II keratin that plays a vital role in the epidermalization of the squamous epithelium, particularly in cell migration, notably keratinocytes. Silencing *KRT6A* has been demonstrated to inhibit invasion and metastasis in nasopharyngeal cancers [34]. However, its role in lung adenocarcinoma is somewhat ambiguous, with varying reports suggesting that it may act as both an oncogene and a tumor suppressor [34,35].

Elevated levels of keratin 6A in lung adenocarcinoma have been associated with a poorer patient prognosis, promoting growth and metastasis [34,36]. Knocking down keratin 6A has been found to inhibit cell proliferation, migration, and colony formation ability while not significantly impacting cell viability in lung adenocarcinoma cell cultures. Additionally, it has been suggested that keratin 6A may play a role in promoting the transformation of cancer stem cells (CXCR4high/CD133high) and the epithelial–mesenchymal transition (EMT) [36].

In this study, the expression of *KRT5* was found to be higher in SCC than in ADC. The activity of keratin 5 can be regulated through epigenetic mechanisms. It has been demonstrated that the downregulation of this gene in NSCLC could result from the binding of miR-520a-5p, and, conversely, the expression of miR-520a-5p may be directly regulated through binding with hsa_circ_0017620 [37]. *KRT5* has been widely used as a component of diagnostic panels in Non-Differentiated Respiratory Papillomatosis (NDRP). Keratin 5 was also a component of the combined genetic and epigenetic model with potential therapeutic significance. The model involved three lncRNAs (*LINC00968*, *lnc-FAM92A-9*, and *lnc-PTGFR-1*) and six mRNAs (*CTCFL*, *KRT5*, *LY6D*, *TMEM*, *GBP6*, and *TMEM179*) [38]. Statistically significant differences were also observed in the expression of two immunomarkers, including keratin 5 (AGR2 and KRT5) in ADC and SCC, both at the mRNA and protein levels [39]. AGR2 demonstrated a 97.0% sensitivity and 94.4% specificity in detecting ADC in the training set, while KRT5 showed a 93.9% sensitivity and 98.9% specificity in identifying SCC. The accuracy of the AGR2-KRT5 combination reached 93.3% for ADC, 92.0% for SCC, and 92.6% overall. In the validation cohort, the predictive accuracy of AGR2-KRT5 was up to 100% for ADC and 86.7% for SCC [39]. A proposed five-gene diagnostic signature, including *KRT5*, *MUC1*, *TREM1*, *C3*, and *TMPRSS2* [40], as well as an eight-gene classifier for lung cancer subtypes [41], exhibited a high predictive accuracy of 0.936 during testing on 2556 adenocarcinoma samples and 1630 squamous cell carcinoma samples. Additionally, the signature performed well in clinically challenging cases, such as poorly differentiated tumors and samples obtained from biopsies [41]. Keratin 5 was also analyzed as part of a panel differentiating ADC from SCC, comprising 10 miRNAs and 10 mRNAs (let-7a-5p, miR-338, miR-375, miR-217, miR-627, miR-140, miR-147b, miR-138-2, miR-584, and miR-197; *CLDN3*, *DSG3*, *KRT17*, *TMEM125*, *KRT5*, *NKX2-1*, *KRT7*, *ABCC5*, *KRAS*, and *PLCG2*). Analyses revealed that *KRT5* is a strong risk factor for ADC and may be silenced by let-7a-5p [42].

Our study also revealed the increased expression of *KRT6C* in SCC compared to ADC. Existing research indicates that *circ-KRT6C* is overexpressed in colorectal cancer tissues and cells, and it is associated with overall patient survival. In colorectal cancer, silencing *circ-KRT6C* has been shown to inhibit growth, migration, invasion, and immune evasion while promoting apoptosis in cancer cells, particularly when miR-485-3p is deficient. *Circ-KRT6C* has been found to increase PDL1 expression by acting as a “sponge” for miR-485-3p, thereby enhancing the malignancy of colorectal cancer cells [43].

The study further identified significantly higher expression of *SERPINB5* in SCC compared to ADC. *SERPINB5* has been implicated in an oncogenic role in gastric cancer. It interacts with KHDRBS3 and FBXO32, where KHDRBS3 potentially influences *FBXO32* mRNA [44]. In pancreatic ductal adenocarcinoma (PDAC), *SERPINB5* appears to function as an oncogene, and unmethylated *SERPINB5* has been identified as a specific marker for PDAC in clinical samples, suggesting its potential utility for liquid biopsies [45]. Existing research in the field of lung cancer has indicated that the high expression of specific genes, including *COL1A2*, *POSTN*, *DSG2*, *CDKN2A*, *COL1A1*, *SLC2A1*, *SERPINB5*, and *SPP1*, can facilitate the diagnosis of NSCLC and correlate with poorer patient outcomes [46]. Moreover, a panel consisting of eight genes associated with the extracellular matrix (*FERMT1*, *CTSV*, *CPS1*, *ENTPD2*, *SERPINB5*, *ITGA8*, *ADAMTS8*, and *LYPD3*) has been constructed, which has been linked to shorter survival, worse immunological outcomes, and higher tumor purity in lung adenocarcinoma patients [47].

Notably, *DSG3* emerged as another classifier among the analyzed molecules. These findings align with previous functional studies highlighting the significant role of *DSG3* in tumor progression, particularly in differentiating adenocarcinoma (ADC) from squamous cell carcinoma (SCC) [48,49].

Our diagnostic models are inherently predictive in nature, as they serve to discern specific NSCLC subtypes, thereby influencing the eligibility of patients for personalized therapeutic interventions. Consequently, the models indirectly prognosticate the efficacy of the prescribed treatment. The expression profiles we meticulously analyzed notably unveil a conspicuous association with the histological subtype of NSCLC, irrespective of concurrent clinical factors. Consequently, our devised diagnostic models for NSCLC subtyping distinctly eschew correlation with clinicopathological features beyond the cancer subtype. This substantiates the efficacy of these models in precisely delineating the NSCLC subtype, independently of variables such as sex, age, stage, and disease progression. In the epoch of personalized therapies, these models exhibit considerable practicality for both neo- and adjuvant therapy, thereby underscoring the models’ extensive applicability and efficacy.

Our findings represent a significant step forward in tailoring therapeutic strategies and guiding drug development for NSCLC. They help identify personalized treatment options, crucial for optimizing preoperative targeted interventions, known as neoadjuvant therapies. These therapies involve administering drugs before surgery to shrink tumors and improve surgical outcomes. Neoadjuvant therapies can be used alone or alongside conventional chemotherapy or advanced immunotherapy. Clinical trials, including NEOSTAR, LCMC3, and NADIM, are evaluating the effectiveness and safety of these interventions across various NSCLC subtypes and molecular profiles [50,51,52].

Our study aimed to uncover the prognostic implications of gene expression profiles in NSCLC patients following surgery. Building on the idea that the molecular characteristics of cancer cells may predict tumor progression, we compared patients who experienced recurrence within 3 years with those who did not. However, we found no statistically significant genes within our study cohort. Notably, previous research has mainly focused on overall survival as the primary endpoint, which has limited sensitivity in prognostic test development. Therefore, our study seeks to overcome this limitation by correlating mRNA expression levels with the progression status of NSCLC patients.

In our investigation, we conducted a comprehensive analysis of gene expression, focusing specifically on selected genes in NSCLC. Nevertheless, it is crucial to acknowledge certain limitations inherent in our approach. Firstly, our decision to exclusively compare gene expressions in tumors, considering both validated and reference genes without incorporating healthy tissue comparisons, was made to prioritize subtype differentiation. However, this choice may have inadvertently limited our insights into the broader cancer biology, as it omitted potential comparisons with healthy tissues. Additionally, the gene MIR205HG, a pivotal focus in our study, exhibits variations across various cancer types and even within lung squamous cell carcinoma [30,31]. This variability poses challenges for its clinical application as a diagnostic marker. Another gene of interest, KRT6A, demonstrated significantly higher expression in squamous cell carcinoma compared to adenocarcinoma. However, its role in lung adenocarcinoma remains unclear, with conflicting reports on whether it acts as an oncogene or a tumor suppressor [34,35]. This ambiguity affects its utility as a diagnostic marker. Our study primarily concentrated on the validation of gene expression without delving into the functional mechanisms of these genes in different lung cancer subtypes. This limitation hinders a comprehensive understanding of the underlying biology. Furthermore, the gene panel we analyzed was relatively small. Expanding it to include more genes associated with lung cancer subtypes could potentially enhance the accuracy of subtype differentiation. Given the high heterogeneity of lung cancer, our study may not have captured all the nuances associated with its subtypes. Although our model demonstrated strong performance in differentiating lung cancer subtypes, incorporating additional clinical and molecular factors has the potential to further improve subtype classification accuracy. Future research efforts should consider these limitations and aim for a more comprehensive exploration of the molecular landscape of lung cancer. In the near future, we aim to investigate subtype differentiation in advanced NSCLC patients, focusing on cases where diagnostic material from biopsies or bronchoscopies is limited. We will analyze the mutation profiles of clinically relevant genes in cases labeled as “not otherwise specified” (NOS) and subtype them using our model. Comparing these results with NOS cases carrying characteristic mutations for non-squamous-cell carcinoma (noSqCC) will demonstrate the effectiveness of our diagnostic tool.

In summary, our study highlights the potential of specific genes in distinguishing lung cancer subtypes, regardless of clinical characteristics. While the analyzed genes did not correlate with NSCLC progression, their statistical significance supports their role as diagnostic markers. Despite methodological differences, our findings emphasize the importance of MIR205HG, KRT5, KRT6A, KRT6C, SERPINB5, and DSG3 in subtype differentiation. Both the gradient boosting decision tree and logistic regression models show effectiveness, each with unique strengths. This underscores the need to integrate molecular insights into clinical practice for accurate subtype identification, particularly in challenging histological cases.

## 4. Materials and Methods

The research was carried out under the auspices of the Polish STRATEGMED-2 initiative, using snap-frozen tumors acquired from non-small-cell lung cancer (NSCLC) patients at the Department of Thoracic Surgery, Medical University of Bialystok. Written informed consent was obtained from all patients for specimen collection and the processing of clinicopathological data. The study’s protocol was approved by the Bioethics Committee of the Medical University of Bialystok, under ethical approval code: R-I-002/357/2014.

### 4.1. Patients and Samples

The study employed a group of 140 surgically excised instances of non-small-cell lung cancer (NSCLC). Inclusion criteria comprised an original diagnosis of lung adenocarcinoma (ADC) or squamous cell carcinoma (SCC) based on histologic evidence, complete resection of the tumor with free margins, stage IA to IIIA, a minimum of 3-year follow-up with monitoring for cancer recurrence events, availability of representative fresh-frozen tumor specimens (with at least 50% tumor cells for RNA extraction), and no history of neoadjuvant chemotherapy or radiotherapy. All procedures related to patient selection for the study and the study design adhered strictly to standard operating procedures, which were meticulously maintained throughout the experiment’s duration [53,54].

### 4.2. Histopathological Diagnosis

Each tumor sample underwent meticulous histopathological evaluation following WHO lung cancer classification, guided by IASLC/ATS/ERS guidelines [55,56]. Immunohistochemical staining resolved diagnostic uncertainties by assessing markers for adenocarcinoma (TTF-1) and squamous cell carcinoma (p63). This approach ensured sample subtype confirmation and RNA content sufficiency, highlighting methodological precision and reliability.

### 4.3. RNA Extraction and Quality Control

Total RNA was extracted from freshly frozen tumor tissues employing the mirVana miRNA Isolation Kit (Ambion, Austin, TX, USA) in accordance with the manufacturer’s guidelines. The concentration, quality, and purity of the resultant total RNA solution underwent comprehensive assessment through both spectrophotometric and microcapillary electrophoresis methods. Initially, the UV/VIS spectrophotometer NanoDrop 2000c (Thermo Scientific, Wilmington, DE, USA) was utilized to ascertain the quantity and purity of the RNA. Subsequently, the integrity necessary for gene expression analysis (RNA Integrity Number above 7) was determined for the isolated total RNA, employing the Agilent RNA 6000 Nano Kit on the Bioanalyzer 2100 (Agilent Technologies, Santa Clara, CA, USA). The analyses conducted facilitated the selection of RNA solutions that met the criteria of a minimum concentration of 100 ng/µL and a RIN value of at least 7. This ensured their suitability for progression to subsequent phases of research.

### 4.4. Quantitative Real-Time PCR Analysis

The reverse transcription (RT) reaction was executed using the High-Capacity RNA-to-cDNA Master Mix with the No RT Control kit (Applied Biosystems, Foster City, CA, USA). This validation study aimed to assess the expression levels of six designated genes (*KRT5*, *KRT6A*, *KRT6C*, *SERPINB5*, *MIR205HG*, and *DSG3*) as potential candidates for precise differentiation between squamous cell carcinoma (SCC) and adenocarcinoma (ADC) in non-small-cell lung cancer (NSCLC). The reference gene utilized for normalization was *18S rRNA*. The selection of these specific genes was informed by prior gene expression microarray analyses conducted at the Department of Clinical Molecular Biology, Medical University of Bialystok, with the results published in 2017 [17]. 

The expression levels of six genes and a reference gene (*18S rRNA*) were assessed using quantitative real-time PCR (qRT-PCR) with commercially validated assays (see Table 4). The qPCR reactions were conducted in triplicates on the ABI PRISM 7900HT instrument (Applied Biosystems, Foster City, USA) following the manufacturer’s protocol (TaqMan Gene Expression Assays Protocol). Raw data (Ct values) were normalized using the formula: ∆Ct = Ct gene − Ct reference gene (*18S rRNA*), yielding Relative Quantification values (RQ).

### 4.5. Statistical Analysis

The normality of ∆Ct value distribution was assessed through the Shapiro–Wilk test. To identify statistically significant differences in ∆Ct values for the studied genes between SCC and ADC histologies, as well as recurrence status, and considering other clinical-pathological parameters, we employed the non-parametric U Mann–Whitney test. These analyses were performed using Statistica 13.3 software. Employing a logistic regression model and a gradient boosting decision tree classifier facilitated the determination of the diagnostic value of the analyzed variables in distinguishing histological subtypes of lung cancer into SCC and ADC. In a total of 100 random assignments, samples were allocated to training and test sets in a 3 to 7 ratio, ensuring balanced classes within each set. Throughout cross-validation, various performance metrics, including receiver operating characteristic (ROC) curves, area under the ROC (AUC), accuracy, recall, sensitivity, F1 score, specificity, precision, and negative predictive value (NPV), were computed. The findings were presented as average values, accompanied by corresponding 95% confidence intervals (CIs). To visualize the decision boundary, six logistic regression models were independently created for each gene. The construction and evaluation of the models involved the use of the following specific Python packages: scikit-learn v1.0.2 [57] for logistic regression and model assessment, and xgboost v1.7.6 [58] for the gradient boosting decision tree classifier. To delve deeper into the biological distinctions between SCC and ADC, we conducted Gene Ontology (GO) analysis. This analytical approach categorizes genes based on their involvement in biological processes, molecular functions, and cellular components. Our analysis aimed to determine if mRNA target genes associated with specific GO terms were significantly over-represented in one group compared to the other. We considered GO terms with a *p*-value of less than 0.01 as statistically significant. It is worth noting that all database accesses occurred in 1 February 2024 (https://geneontology.org/).

The assessment of ∆Ct value distribution for normality was conducted through the Shapiro–Wilk test. To discern statistically significant disparities in ∆Ct values among the studied genes across SCC and ADC histologies, as well as recurrence status and other clinical–pathological parameters, the non-parametric U Mann–Whitney test was employed. These analyses were executed using Statistica 13.3 software.

Utilizing a logistic regression model and a gradient boosting decision tree classifier facilitated the evaluation of the diagnostic efficacy of the analyzed variables in distinguishing between histological subtypes of lung cancer, namely, SCC and ADC. In a total of 100 random assignments, samples were divided into training and test sets in a 3:7 ratio to ensure balanced classes within each set. Throughout cross-validation, various performance metrics, including receiver operating characteristic (ROC) curves, area under the ROC (AUC), accuracy, recall, sensitivity, F1 score, specificity, precision, and negative predictive value (NPV), were computed. The results were presented as mean values, accompanied by corresponding 95% confidence intervals (CIs).

For visualizing the decision boundary, six logistic regression models were independently generated for each gene. The development and evaluation of these models utilized the following specific Python packages: scikit-learn v1.0.2 [57] for logistic regression and model assessment, and xgboost v1.7.6 [58] for the gradient boosting decision tree classifier.

To delve deeper into the biological distinctions between SCC and ADC, Gene Ontology (GO) analysis was conducted. This analytical approach categorizes genes based on their involvement in biological processes, molecular functions, and cellular components. Our analysis aimed to ascertain whether mRNA target genes associated with specific GO terms were significantly over-represented in one group compared to the other. GO terms with a *p*-value of less than 0.01 were considered statistically significant. It is noteworthy that all database accesses occurred in 1 February 2024 (https://geneontology.org/).

## 5. Conclusions

In this study, we developed robust models capable of distinguishing between different subtypes of non-small-cell lung cancer, enhancing the accuracy of existing diagnostic protocols. By validating the expression profiles of six specific genes (MIR205HG, KRT5, KRT6A, KRT6C, SERPINB5, and DSG3), selected based on a previously established 53-gene signature, we consistently observed higher expression levels in squamous cell carcinoma (SCC) compared to adenocarcinoma (ADC). However, these genes did not correlate with the progression status of NSCLC patients. Using advanced computational techniques, we found significant differences in gene expression between SCC and ADC, with strong predictive value demonstrated by both the gradient boosting decision tree and logistic regression classifiers. Notably, while most gene sets showed robust predictive capabilities, KRT6C alone exhibited diminished efficacy, highlighting the importance of complementary genes. Our study underscores the potential of these genes as independent discriminative factors for NSCLC histopathology, regardless of clinical factors. The statistical significance observed reaffirms the utility of examining gene expression profiles in understanding the molecular landscape of lung cancer subtypes. Further scrutiny and the functional annotation of these genes are necessary before their integration into clinical practice. Overall, our findings contribute to advancing our understanding of molecular changes in lung cancer, particularly in deciphering the complex gene expression dynamics underlying histological variability in NSCLC.

## Figures and Tables

**Table 1:** Patients’ characteristics.

Gender	N	%
Male	107	76.43
Female	33	23.57
Age at Diagnosis (years)		
Range	40–81	
Mean	63.33	
Histological Type		
Squamous cell carcinoma	79	56.42
Adenocarcinoma	61	43.57
TNM Stage		
IA	33	23.57
IB	32	22.86
IIA	28	20.0
IIB	38	27.14
IIIA	8	5.71
Information not available	1	0.7
Recurrence		
Present	54	38.57
Absent	82	58.57
Information not available	4	2.86

**Table 2:** Expression of six validated genes in SCC tumors vs. ADC tumors. ∆Ct values are inversely proportional to the direction of relative gene expression.

Genes ∆Ct SCC						Genes ∆Ct ADC						Expression Profile
	N	Mean	SD	25%	75%	N	Mean	SD	25%	75%	*p*-Value	SCC vs. ADC
*MIR-205HG*	81	14.808	2.769	13.226	15.362	59	20.099	3.426	17.940	22.425	<0.001	up
*SERPINB5*	81	14.850	2.990	12.725	15.977	59	20.305	3.640	18.546	22.048	<0.001	up
*KRT6C*	81	16.697	3.104	14.670	18.749	59	18.422	3.041	16.061	20.628	0.003381	up
*KRT6A*	81	11.574	3.145	9.580	13.001	59	16.578	2.858	14.772	19.071	<0.001	up
*KRT5*	81	11.968	3.492	9.543	12.405	59	19.383	3.369	17.277	21.823	<0.001	up
*DSG*	81	14.864	4.012	12.172	16.386	59	23.050	3.559	21.261	25.255	<0.001	up

**Table 3:** Gene Ontology analyses.

GO Category	GO Term Id	GO Term Label	*p*-Value	Genes Involved
Cellular component	GO:0001533	cornified envelope	8.86 × 10^−5^	*DSG3, SERPINB5*
	GO:0045095	keratin filament	1.10 × 10^−6^	*KRT6A, KRT5, KRT6C*
	GO:0005882	intermediate filament	1.35 × 10^−5^	*KRT6A*
	GO:0045111	intermediate filament cytoskeleton	2.09 × 10^−5^	*KRT6A, KRT5, KRT6C*
	GO:0005615	extracellular space	0.00011	*DSG3, KRT6A, KRT5, KRT6C, SERPINB5*
Biological process	GO:0045109	intermediate filament organization	4.99 × 10^−7^	*KRT6A, KRT5, KRT6C*
	GO:0045104	intermediate filament cytoskeleton organization	1.04 × 10^−6^	*KRT6A, KRT5, KRT6C*
	GO:0045103	intermediate filament-based process	1.07 × 10^−6^	*KRT6A, KRT5, KRT6C*
	GO:0031424	keratinization	6.99 × 10^−7^	*KRT6A, KRT5, KRT6C*
	GO:0030216	keratinocyte differentiation	3.67 × 10^−6^	*KRT6A, KRT5, KRT6C*
	GO:0009913	epidermal cell differentiation	1.10 × 10^−5^	*KRT6A, KRT5, KRT6C*
Molecular function	GO:0030280	structural constituent of skin epidermis	6.77 × 10^−8^	*KRT6A, KRT5, KRT6C*

**Table 4:** Validated genes as candidates for histotypic discrimination between SCC and ADC.

RefSeq	Gene Name, Abbreviation	Assay IDs from Applied Biosystems (Primers + Probe)
NM_000424.3	Keratin 5 (*KRT5*)	Hs00361185_m1
NM_005554.3	Keratin 6A (*KRT6A*)	Hs01699178_g1
NM_002639.4 XM_006722483.2	Serpin Family B Member 5 (*SERPINB5*)	Hs00985285_m1
NM_001104548.1 XM_017002058.1	MIR205 Host Gene (*MIR205HG*)	Hs03405498_m1
NM_173086.4	Keratin 6C (*KRT6C*)	Hs00752476_s1
NM_001944.2 XM_011525850.1	Desmoglein 3 (*DSG3*)	Hs00951897_m1
X03205.1	*18S rRNA* (Reference Gene)	Hs99999901_s1

## Data Availability

The datasets analyzed during the current study are available from the corresponding author on reasonable request.

## Supplementary Materials

The supporting information can be downloaded at: https://www.mdpi.com/article/10.3390/ijms25073607/s1.

## References

[1] Sulewska A., Pilz L., Manegold C., Ramlau R., Charkiewicz R., Niklinski J. (2023). A Systematic Review of Progress toward Unlocking the Power of Epigenetics in NSCLC: Latest Updates and Perspectives. Cells.

[2] Charkiewicz R., Pilz L., Sulewska A., Kozlowski M., Niklinska W., Moniuszko M., Reszec J., Manegold C., Niklinski J. (2016). Validation for histology-driven diagnosis in non-small cell lung cancer using hsa-miR-205 and hsa-miR-21 expression by two different normalization strategies. Int. J. Cancer.

[3] Tane S., Nishio W., Ogawa H., Hokka D., Tane K., Tanaka Y., Tauchi S., Uchino K., Sakai Y., Ohbayashi C. (2014). Clinical significance of the “not otherwise specified” subtype in candidates for resectable non-small cell lung cancer. Oncol. Lett..

[4] Al-Farsi A., Ellis P.M. (2014). Treatment paradigms for patients with metastatic non-small cell lung cancer, squamous lung cancer: First, second, and third-line. Front. Oncol..

[5] Uramoto H., Tanaka F. (2014). Recurrence after surgery in patients with NSCLC. Transl. Lung Cancer Res..

[6] Molina J.R., Yang P., Cassivi S.D., Schild S.E., Adjei A.A. (2008). Non-small cell lung cancer: Epidemiology, risk factors, treatment, and survivorship. Mayo Clin. Proc..

[7] Herbst R.S., Heymach J.V., Lippman S.M. (2008). Lung cancer. N. Engl. J. Med..

[8] Shroff G.S., de Groot P.M., Papadimitrakopoulou V.A., Truong M.T., Carter B.W. (2018). Targeted Therapy and Immunotherapy in the Treatment of Non-Small Cell Lung Cancer. Radiol. Clin. N. Am..

[9] Pal S.K., Figlin R.A., Reckamp K. (2010). Targeted therapies for non-small cell lung cancer: An evolving landscape. Mol. Cancer Ther..

[10] Lauro S., Onesti C.E., Righini R., Marchetti P. (2014). The use of bevacizumab in non-small cell lung cancer: An update. Anticancer Res..

[11] Chen Z., Akbay E., Mikse O., Tupper T., Cheng K., Wang Y., Tan X., Altabef A., Woo S.-A., Chen L. (2014). Co-clinical trials demonstrate superiority of crizotinib to chemotherapy in ALK-rearranged non-small cell lung cancer and predict strategies to overcome resistance. Clin. Cancer Res..

[12] Thakur M.K., Wozniak A.J. (2017). Spotlight on necitumumab in the treatment of non-small-cell lung carcinoma. Lung Cancer.

[13] Wu Y.-L., Herbst R.S., Mann H., Rukazenkov Y., Marotti M., Tsuboi M. (2018). ADAURA: Phase III, Double-blind, Randomized Study of Osimertinib Versus Placebo in EGFR Mutation-positive Early-stage NSCLC After Complete Surgical Resection. Clin. Lung Cancer.

[14] Charkiewicz R., Sulewska A., Charkiewicz A., Gyenesei A., Galik B., Ramlau R., Piwkowski C., Stec R., Biecek P., Karabowicz P. (2023). miRNA-Seq Tissue Diagnostic Signature: A Novel Model for NSCLC Subtyping. Int. J. Mol. Sci..

[15] Sulewska A., Niklinski J., Charkiewicz R., Karabowicz P., Biecek P., Baniecki H., Kowalczuk O., Kozlowski M., Modzelewska P., Majewski P. (2022). A Signature of 14 Long Non-Coding RNAs (lncRNAs) as a Step towards Precision Diagnosis for NSCLC. Cancers.

[16] Charkiewicz R., Sulewska A., Mroz R., Charkiewicz A., Naumnik W., Kraska M., Gyenesei A., Galik B., Junttila S., Miskiewicz B. (2023). Serum Insights: Leveraging the Power of miRNA Profiling as an Early Diagnostic Tool for Non-Small Cell Lung Cancer. Cancers.

[17] Charkiewicz R., Niklinski J., Claesen J., Sulewska A., Kozlowski M., Michalska-Falkowska A., Reszec J., Moniuszko M., Naumnik W., Niklinska W. (2017). Gene Expression Signature Differentiates Histology but Not Progression Status of Early-Stage NSCLC. Transl. Oncol..

[18] Tibshirani R., Hastie T., Narasimhan B., Chu G. (2002). Diagnosis of multiple cancer types by shrunken centroids of gene expression. Proc. Natl. Acad. Sci. USA.

[19] Li D., Yang W., Zhang Y., Yang J.Y., Guan R., Xu D., Yang M.Q. (2018). Genomic analyses based on pulmonary adenocarcinoma in situ reveal early lung cancer signature. BMC Med. Genom..

[20] Niemira M., Collin F., Szalkowska A., Bielska A., Chwialkowska K., Reszec J., Niklinski J., Kwasniewski M., Kretowski A. (2020). Molecular Signature of Subtypes of Non-Small-Cell Lung Cancer by Large-Scale Transcriptional Profiling: Identification of Key Modules and Genes by Weighted Gene Co-Expression Network Analysis (WGCNA). Cancers.

[21] Song J.-Y., Li X.-P., Qin X.-J., Zhang J.-D., Zhao J.-Y., Wange R. (2020). A fourteen-lncRNA risk score system for prognostic prediction of patients with non-small cell lung cancer. Cancer Biomark..

[22] Girard L., Rodriguez-Canales J., Behrens C., Thompson D.M., Botros I.W., Tang H., Xie Y., Rekhtman N., Travis W.D., Wistuba I.I. (2016). An Expression Signature as an Aid to the Histologic Classification of Non-Small Cell Lung Cancer. Clin. Cancer Res..

[23] Hou J., Aerts J., den Hamer B., van Ijcken W., den Bakker M., Riegman P., Van Der Leest C., Van Der Spek P., Foekens J.A., Hoogsteden H.C. (2010). Gene expression-based classification of non-small cell lung carcinomas and survival prediction. PLoS ONE.

[24] Wilkerson M.D., Schallheim J.M., Hayes D.N., Roberts P.J., Bastien R.R.L., Mullins M., Yin X., Miller C.R., Thorne L.B., Geiersbach K.B. (2013). Prediction of lung cancer histological types by RT-qPCR gene expression in FFPE specimens. J. Mol. Diagn..

[25] Lu C., Chen H., Shan Z., Yang L. (2016). Identification of differentially expressed genes between lung adenocarcinoma and lung squamous cell carcinoma by gene expression profiling. Mol. Med. Rep..

[26] Dong X., Chen X., Lu D., Diao D., Liu X., Mai S., Feng S., Xiong G. (2021). LncRNA miR205HG hinders HNRNPA0 translation: Anti-oncogenic effects in esophageal carcinoma. Mol. Oncol..

[27] Zhang W., Liang F., Li Q., Sun H., Li F., Jiao Z., Lei J. (2022). LncRNA MIR205HG accelerates cell proliferation, migration and invasion in hepatoblastoma through the activation of MAPK signaling pathway and PI3K/AKT signaling pathway. Biol. Direct.

[28] Yin L., Zhang Y., Zheng L. (2022). Analysis of differentially expressed long non-coding RNAs revealed a pro-tumor role of MIR205HG in cervical cancer. Mol. Med. Rep..

[29] Di Agostino S., Valenti F., Sacconi A., Fontemaggi G., Pallocca M., Pulito C., Ganci F., Muti P., Strano S., Blandino G. (2018). Long Non-coding MIR205HG Depletes Hsa-miR-590-3p Leading to Unrestrained Proliferation in Head and Neck Squamous Cell Carcinoma. Theranostics.

[30] Zhao X., Yuan C., He X., Wang M., Zhang H., Cheng J., Wang H. (2022). Identification and in vitro validation of diagnostic and prognostic biomarkers for lung squamous cell carcinoma. J. Thorac. Dis..

[31] Liu L., Li Y., Zhang R., Li C., Xiong J., Wei Y. (2020). MIR205HG acts as a ceRNA to expedite cell proliferation and progression in lung squamous cell carcinoma via targeting miR-299-3p/MAP3K2 axis. BMC Pulm. Med..

[32] Chang Y., Xue X., Li C., Zhao W., Ma Y., Xu F., Wu Z., Dai Y., Li Y., Liu Y. (2020). MIR205HG facilitates carcinogenesis of lung squamous cell carcinoma in vitro revealed by long noncoding RNA profiling. Acta Biochim. Biophys. Sin..

[33] Larzabal L., de Aberasturi A.L., Redrado M., Rueda P., Rodriguez M.J., Bodegas M.E., Montuenga L.M., Calvo A. (2014). TMPRSS4 regulates levels of integrin α5 in NSCLC through miR-205 activity to promote metastasis. Br. J. Cancer.

[34] Che D., Wang M., Sun J., Li B., Xu T., Lu Y., Pan H., Lu Z., Gu X. (2021). KRT6A Promotes Lung Cancer Cell Growth and Invasion through MYC-Regulated Pentose Phosphate Pathway. Front. Cell Dev. Biol..

[35] Xiao J., Kuang X., Dai L., Zhang L., He B. (2020). Anti-tumour effects of Keratin 6A in lung adenocarcinoma. Clin. Respir. J..

[36] Yang B., Zhang W., Zhang M., Wang X., Peng S., Zhang R. (2020). KRT6A Promotes EMT and Cancer Stem Cell Transformation in Lung Adenocarcinoma. Technol. Cancer Res. Treat..

[37] Chen S., Hong K., Zhou L., Ran R., Huang J., Zheng Y., Xing M., Cai Y. (2022). Hsa_circRNA_0017620 regulated cell progression of non-small-cell lung cancer via miR-520a-5p/KRT5 axis. J. Clin. Lab. Anal..

[38] Xiao K., Wang Y., Zhou L., Wang J., Wang Y., Tong D., Zhu Z., Jiang J. (2021). Construction of ceRNA network to identify the lncRNA and mRNA related to non-small cell lung cancer. PLoS ONE.

[39] Pan B., Wei Z.-X., Zhang J.-X., Li X., Meng Q.-W., Cao Y.-Y., Qi L.-S., Yu Y. (2021). The value of AGR2 and KRT5 as an immunomarker combination in distinguishing lung squamous cell carcinoma from adenocarcinoma. Am. J. Transl. Res..

[40] Wu X., Wang L., Feng F., Tian S. (2020). Weighted gene expression profiles identify diagnostic and prognostic genes for lung adenocarcinoma and squamous cell carcinoma. J. Int. Med. Res..

[41] Hamaneh M., Yu Y.-K. (2022). An 8-Gene Signature for Classifying Major Subtypes of Non-Small-Cell Lung Cancer. Cancer Inform..

[42] Yu H., Pang Z., Li G., Gu T. (2021). Bioinformatics analysis of differentially expressed miRNAs in non-small cell lung cancer. J. Clin. Lab. Anal..

[43] Jiang Z., Hou Z., Liu W., Yu Z., Liang Z., Chen S. (2021). circ-Keratin 6c Promotes Malignant Progression and Immune Evasion of Colorectal Cancer through microRNA-485-3p/Programmed Cell Death Receptor Ligand 1 Axis. J. Pharmacol. Exp. Ther..

[44] Lei K.-F., Liu B.-Y., Wang Y.-F., Chen X.-H., Yu B.-Q., Guo Y., Zhu Z.-G. (2011). SerpinB5 interacts with KHDRBS3 and FBXO32 in gastric cancer cells. Oncol. Rep..

[45] Mardin W.A., Ntalos D., Mees S.T., Spieker T., Senninger N., Haier J., Dhayat S.A. (2016). SERPINB5 Promoter Hypomethylation Differentiates Pancreatic Ductal Adenocarcinoma from Pancreatitis. Pancreas.

[46] Su C., Liu W.-X., Wu L.-S., Dong T.-J., Liu J.-F. (2021). Screening of Hub Gene Targets for Lung Cancer via Microarray Data. Comb. Chem. High Throughput Screen..

[47] Xiao L., Li Q., Huang Y., Fan Z., Qin W., Liu B., Yuan X. (2022). Integrative Analysis Constructs an Extracellular Matrix-Associated Gene Signature for the Prediction of Survival and Tumor Immunity in Lung Adenocarcinoma. Front. Cell Dev. Biol..

[48] Abula Y., Su Y., Tuniyazi D., Yi C. (2021). Desmoglein 3 contributes to tumorigenicity of pancreatic ductal adenocarcinoma through activating Src-FAK signaling. Anim. Cells Syst..

[49] Dong Y., Li S., Sun X., Wang Y., Lu T., Wo Y., Leng X., Kong D., Jiao W. (2020). Desmoglein 3 and Keratin 14 for Distinguishing Between Lung Adenocarcinoma and Lung Squamous Cell Carcinoma. Onco Targets Ther..

[50] Sepesi B., Mehran R., Spicer J., Cascone T. (2023). NEOSTAR trial and the current status of neoadjuvant therapy in non-small cell lung cancer. J. Thorac. Cardiovasc. Surg..

[51] Chen L.N., Wei A.Z., Shu C.A. (2023). Neoadjuvant immunotherapy in resectable non-small-cell lung cancer. Ther. Adv. Med. Oncol..

[52] Provencio M., Nadal E., Insa A., García-Campelo M.R., Casal-Rubio J., Dómine M., Majem M., Rodríguez-Abreu D., Martínez-Martí A., Carpeño J.D.C. (2020). Neoadjuvant chemotherapy and nivolumab in resectable non-small-cell lung cancer (NADIM): An open-label, multicentre, single-arm, phase 2 trial. Lancet Oncol..

[53] Niklinski J., Kretowski A., Moniuszko M., Reszec J., Michalska-Falkowska A., Niemira M., Ciborowski M., Charkiewicz R., Jurgilewicz D., Kozlowski M. (2017). Systematic biobanking, novel imaging techniques, and advanced molecular analysis for precise tumor diagnosis and therapy: The Polish MOBIT project. Adv. Med. Sci..

[54] Michalska-Falkowska A., Niklinski J., Juhl H., Sulewska A., Kisluk J., Charkiewicz R., Ciborowski M., Ramlau R., Gryczka R., Piwkowski C. (2023). Applied Molecular-Based Quality Control of Biobanked Samples for Multi-Omics Approach. Cancers.

[55] Travis W., Brambilla E., Muller-Hermelink H., Harris C. (2004). Pathology and Genetics of Tumours of the Lung, Pleura, Thymus and Heart. WHO Classification of Tumours.

[56] Travis W.D., Brambilla E., Noguchi M., Nicholson A.G., Geisinger K.R., Yatabe Y., Beer D.G., Powell C.A., Riely G.J., Van Schil P.E. (2011). International association for the study of lung cancer/american thoracic society/european respiratory society international multidisciplinary classification of lung adenocarcinoma. J. Thorac. Oncol..

[57] Pedregosa F., Varoquaux G., Gramfort A., Michel V., Thirion B. (2011). Scikit-learn: Machine Learning in Python. J. Mach. Learn. Res..

[58] Chen T., Guestrin C. XGBoost: A Scalable Tree Boosting System. Proceedings of the 22nd ACM SIGKDD International Conference on Knowledge Discovery and Data Mining.

